# Cardiac and Inflammatory Necrotizing Enterocolitis in Newborns Are Not the Same Entity

**DOI:** 10.3389/fped.2020.593926

**Published:** 2021-01-06

**Authors:** Michaela Klinke, Hanna Wiskemann, Benjamin Bay, Hans-Jörg Schäfer, Laia Pagerols Raluy, Konrad Reinshagen, Deirdre Vincent, Michael Boettcher

**Affiliations:** ^1^Department of Pediatric Surgery, University Medical Center Hamburg-Eppendorf, Hamburg, Germany; ^2^Department of Cardiology, University Heart and Vascular Center Hamburg-Eppendorf, Hamburg, Germany; ^3^Department of Pathology, University Medical Center Hamburg-Eppendorf, Hamburg, Germany

**Keywords:** necrotizing enterocolitis, NEC, neonates, NETs, neutrophil extracellular traps, cardiology

## Abstract

**Background:** Necrotizing enterocolitis (NEC) is an often-fatal neonatal disease involving intestinal hyperinflammation leading to necrosis. Despite ongoing research, (1) conflicting results and (2) comorbidities of NEC patients make early NEC detection challenging and may complicate therapy development. Most research suggests that NEC pathogenesis is multifactorial, involving a combination of (1) gut prematurity; (2) abnormal bacterial colonization; and (3) ischemia-reperfusion (I/R) injury. As neutrophil extracellular traps (NETs) partially mediate I/R injury and drive inflammation in NEC, we hypothesized that NETs contribute to NEC development; particularly in cardiac patients.

**Methods:** A retrospective analysis of baseline characteristics, clinical signs, laboratory parameters, and imaging was conducted for surgically verified NEC cases over 10 years. Patients were stratified into two groups: (1) prior medically or surgically treated cardiac disease (cardiac NEC) and (2) no cardiac comorbidities (inflammatory NEC). Additionally, histology was reassessed for neutrophil activation and NETs formation.

**Results:** A total of 110 patients (cNEC 43/110 vs. iNEC 67/110) were included in the study, with cNEC neonates being significantly older than iNEC neonates (*p* = 0.005). While no significant differences were found regarding clinical signs and imaging, laboratory parameters revealed that cNEC patients have significantly increased leucocyte (*p* = 0.024) and neutrophil (*p* < 0.001) counts. Both groups also differed in pH value (*p* = 0.011). Regarding histology: a non-significant increase in staining of myeloperoxidase within the cNEC group could be found in comparison to iNEC samples. Neutrophil elastase (*p* = 0.012) and citrullinated histone H3 stained (*p* = 0.041) slides showed a significant markup for neonates diagnosed with cNEC in comparison to neonates with iNEC.

**Conclusion:** The study shows that many standardized methods for diagnosing NEC are rather unspecific. However, differing leucocyte and neutrophil concentrations for iNEC and cNEC may indicate a different pathogenesis and may aid in diagnosis. As we propose that iNEC is grounded rather in sepsis and neutropenia, while cNEC primarily involves I/R injuries, which involves neutrophilia and NETs formation, it is plausible that I/R injury due to interventions for cardiac comorbidities results in pronounced neutrophil activation followed by a hyperinflammation reaction and NEC. However, prospective studies are necessary to validate these findings and to determine the accuracy of the potential diagnostic parameters.

## Introduction

Necrotizing enterocolitis (NEC) is one of the most devastating diseases of newborn and preterm infants and has numerous short- and long-term physiological and psychological consequences (1). Despite improvement of neonatal care and the development of novel treatment options, the morbidity and mortality rates of NEC remain high (1). However, due to overall improvements in neonatal care, such as the introduction of donated breast milk, the incidence rate of NEC has been increasing steadily in parallel with rising survival rates of premature infants - the patient population at greatest risk for developing NEC (2–4). One problem contributing to the high mortality rate of NEC patients is the difficulty of diagnosing and treating the condition in a timely manner. Diagnostic methods, such as sonography, abdominal X-ray, and laboratory parameters aid in the diagnosis, but NEC confirmation can only be obtained through surgical exploration (5). As such, gaining an understanding of the NEC pathomechanism is essential in developing and/or finding new diagnostic parameters for NEC diagnosis and, subsequently, achieving better treatment outcomes.

The current consensus on NEC pathogenesis is that NEC is caused by the interaction of a multitude of risk factors, which ultimately lead to a hyperinflammation reaction of the neonatal intestine (6–8). However, most research suggests that the hyperinflammation reaction involves three distinct features: (1) prematurity of the gut; (2) abnormal bacterial colonization, transmigration and inflammation; and (3) ischemia-reperfusion (I/R) injury.

Although, these contributing factors can be described in a clear fashion, the boundaries and interplay between them remains unclear. A distinction of NEC entities based on genesis, however, might aid in the development of more accurate diagnostic criteria and treatments, as well as a more refined understanding of NEC pathogenesis. Thus, based on the initial cause of NEC, a stratification of NEC into: (1) classic inflammatory NEC (iNEC), which occurs mostly in preterms; and (2) cardiac NEC (cNEC), which occurs after medical or surgical intervention for cardiac disease are considered in this study.

The prospect of isolating cNEC from iNEC is based on the consideration that Polin et al. described as early as 1976 that described an increased rate of NEC development in children with congenital heart disease (CHD) (9). This observation can partially be explained by the knowledge that children with CHD either: (1) exhibit a decrease in power of heart contraction or (2) improperly oxygenate blood, resulting in a poorly oxygenated blood supply to the superior mesenteric artery and, consequently, decreased intestinal wall perfusion (10). Upon resolution of the heart problem, the prior decrease in hemodynamics is corrected and blood flow to the previously hypoperfused intestinal area is improved, resulting in an I/R injury of the gut (11, 12). Ultimately, the consensus is that the I/R injury then causes a hyperinflammation reaction through neutrophil activation, gut prematurity, and abnormal intestinal flora, leading to cNEC development (12).

The final result of both iNEC and cNEC is intestinal inflammation, which causes the release of cytokines, allowing for migration of neutrophils to the site of inflammation. Neutrophils are first line responders of the innate immune system and generally combat infections by phagocytosis. However, another more novel infection fighting mechanism employed by neutrophils is the production of neutrophil extracellular traps (NETs), which are web like structures made up of DNA and proteolytic enzymes (13, 14). NETs are cytotoxic, induce thrombosis and aggravate inflammation (aka immunothrombosis) (15, 16). The involvement of NETs in I/R injury and intestinal hyperinflammation including NEC has been shown in previous studies (6, 17, 18). This is particularly evident at high neutrophil densities when NETs tend to become matted and form large aggregates endowed with a plethora of enzymatic activities (aka “aggNETs”), which then accelerate the formation of thrombi in blood vessels (19, 20). Consequently, it is hypothesized that NETs partially mediate the process of I/R injury to the gut in NEC and, therefore, based on our theory, should be found in higher concentrations in neonates with cNEC. Thus, the aim of this retrospective analysis was to assess whether differences amongst neonates diagnosed with iNEC vs. cNEC exist that might aid in a more accurate, reliable, and timely diagnosis of the disease, especially in respect to neutrophils and their role in NEC pathogenesis.

## Methods

### Study Design

The study employed a retrospective analysis of all neonates who underwent surgery for NEC. Files from patients from two Level 1 neonatal intensive care units (Altonaer Children's Hospital, AKK and University Medical Center Hamburg-Eppendorf, UKE) were analyzed during the timeframe of 2007 to 2017. All patients with surgically confirmed NEC diagnosis were included in the study. Patients with (1) previous cardiac surgery or (2) surgical or medical patent ductus arteriosus (PDA) closure prior to NEC surgery were considered cNEC patients. All other patients were assigned to the iNEC group. The following datapoints were assessed for all subjects:

1. General baseline characteristics and common complications of prematurity Patient files were evaluated with respect to the following characteristics to control for potential confounders.
  - Baseline characteristics: (1) Birth weight; (2) gestational age; (3) gender.
  Neonates diagnosed with cNEC also were assessed for the following:
  - Diagnosis: (1) PDA, (2) complex cardiac defects (patients included in this group were diagnosed with one of the following: ventricular septal defects, atrial septal defects II, Fallot tetralogy, interrupted aortic arch type B, pulmonary arteriovenous malformations)
  - Time after birth resp. cardiac intervention.
2. Diagnostic signs
  Diagnostic signs, like laboratory parameters and clinical signs as well as imaging were only included for the current analysis if they were assessed within 8 h before surgery.
  The following laboratory parameters were examined for all individuals enrolled in the study prior to undergoing surgery confirming the NEC diagnosis:
  - Blood Gas Analysis (BGA) Parameters: (1) pH; (2) sodium; (3) base excess; (4) lactate; (5) glucose
  - Clinical chemistry: (1) C-reactive protein (CRP); (2) interleukin-6 (IL-6)
  - Red and white blood cell count: (1) Leucocytes; (2) neutrophils; (3) thrombocytes.
  Clinical symptoms of NEC, as well as imaging findings that are characteristic of NEC, were evaluated in all neonates included in the study.
  - Clinical signs: (1) Bilious gastric fluid; (2) hematochezia; (3) abdominal distension; (4) abdominal rigidity
  - Imaging investigations: (1) Pneumatosis in ultrasound (US); (2) portal venous gas in US; (3) free air in US; (4) pneumatosis in X-Ray (RX); (5) portal venous gas in RX; (6) free air in RX; (7) football sign in RX.

In addition, intestinal histology from the pathology department of the UKE of 24 NEC patients of the current cohort, in whom tissue samples were available, were reassessed. Upon further analysis, based on our classification criteria, 16 of the 24 were considered iNEC patients, while the remaining eight were included in the cNEC group. Immunohistochemical staining for NE and MPO (neutrophil activation and recruitment) and citrullinated Histone H3 (H3cit, NETs formation) were assessed by a pathologist blinded to the study and evaluated as follows:

- None (0)—no signs of tissue staining
- Mild (1)—small amount of tissue staining
- Medium (2)—medium amount of tissue staining
- Severe (3)—large amount of tissue staining.

### Statistics

All data were analyzed using SPSS Statistics 26 (IBM, NY, USA) and GraphPad Prism 8 (GraphPad, CA, USA). Differences between groups were calculated using the *t*-test or Mann-Whitney test for ordinal data. Data are presented as mean ± standard deviation (SD). For association between factors, Spearman's Rho was utilized. For all tests, the level of significance was set at < 0.05.

## Results

During the time period of 2007–2017, 110 patients were diagnosed with NEC, as verified by the attending surgeon at the UKE or AKK. Based on our study criteria, 43 out of the 110 cases (39.1%) fulfilled the criteria for cNEC classification, while the remaining 67 patients were assigned to the iNEC group (60.9%). In the iNEC group 21/67 (31.3%) and in the cNEC group 14/43 (32.6%) patients died (*p* = 0.90). Baseline characteristics, clinical signs, laboratory parameters, and diagnostic investigations are summarized in Table 1.

**Table 1:** Baseline characteristics, clinical signs, laboratory parameters, and diagnostic investigations stratified by patient group assignment of iNEC vs. cNEC.

**Features**	**iNEC (SD) (*n* = 67)**	**cNEC (SD)** **(*n* = 43)**	***p***
Gestational week	28.96 (5.03)	27.55 (4.77)	0.15
Preterm birth (<37 weeks of gestation)	59/67	38/43	1.00
Age at operation (days)	15.91 (14.88)	25.05 (18.23)	**0.005**
Birth weight (g)	1356.99 (874.62)	1,133.95 (886.62)	0.20
Gender (female:male)	37:30	23:20	0.51
Bilious gastric fluid	46/67	33/43	0.39
Hematochezia	19/67	9/43	0.50
Abdominal distension	65/67	40/43	0.37
Abdominal rigidity	6/67	0/43	0.08
BGA pH	7.25 (0.11)	7.19 (0.16)	**0.017**
BGA sodium (mmol/l)	135.90 (7.12)	135.15 (6.78)	0.62
BGA base excess (mmol/l)	−3.38 (6.00)	−4.02 (6.76)	0.60
BGA lactate (mmol/l)	3.69 (3.58)	4.01 (3.90)	0.67
BGA glucose (mg/dl)	139.83 (69.62)	165.71 (73.11)	0.10
LAB leucocytes (10^9^/l)	12.50 (8.02)	16.86 (10.35)	**0.015**
LAB neutrophils (10^9^/l)	5.86 (4.09)	10.73 (4.00)	** <0.001**
LAB thrombocytes (10^9^/l)	216.16 (140.95)	239.59 (199.03)	0.47
LAB CRP (mg/l)	57.32 (73.29)	67.99 (82.97)	0.48
LAB IL-6 (ng/l)	19,089.30 (86,431.66)	14,200.79 (46,773.70)	0.82
US pneumatosis	27/67	20/43	0.63
US portal venous gas	17/67	13/43	0.78
US free air	15/67	5/43	0.34
RX pneumatosis	21/67	16/43	0.67
RX portal venous gas	2/67	2/43	0.63
RX free air	21/67	7/43	0.12
RX football sign	9/67	4/43	0.76

*Bold values differ significantly*.

**Figure 1:**
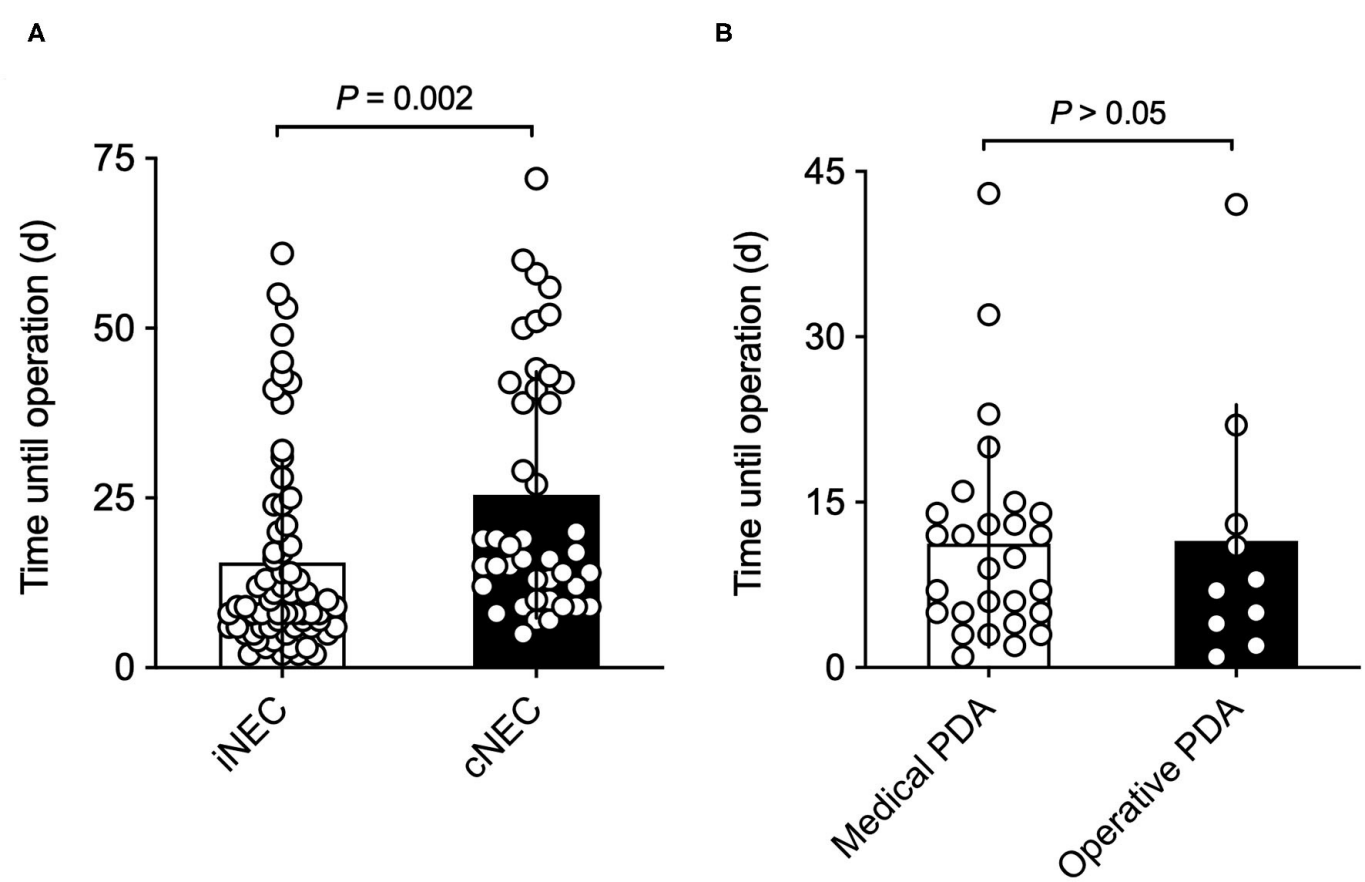
**(A)** Time from birth until NEC operation: neonates with retrospectively diagnosed iNEC were operated on significantly earlier than patients in the cNEC group. **(B)** Time from PDA closure until NEC operation: there are no differences between time of surgically confirmed NEC diagnosis and method of PDA closure. Risk of NEC manifestation is highest in the first 2 weeks of PDA closure.

As shown in Figure 1A, there were significant differences between the patient's age during which the NEC operation took place. Children diagnosed with cNEC were statistically significantly older than neonates diagnosed with iNEC [cNEC 25.05 (SD 18.23) vs. iNEC 15.91 (14.88), *p* = 0.005] but had similar corrected age (31.19 weeks, *p* = 0.82). Upon closer examination of the individuals within the cNEC group undergoing treatment for PDA closure [*n* = 38 (88.4%)], a subgroup analysis revealed that NEC developed within 11.25 (9.38) days post medical PDA closure (mostly indomethacin treatment) vs. 11.50 (12.36) days after operative PDA closure, thus demonstrating no cause-and-effect relationship between type of PDA closure employed and timepoint of NEC development (*p* = 0.95, Figure 1B).

Regarding clinical signs, abdominal distension (iNEC 97% vs. cNEC 93%), followed by feeding intolerance (iNEC 68% vs. cNEC 76%) and hematochezia (iNEC 28%, cNEC 21%) were relatively common but no significant differences were found for both groups (Table 1).

**Figure 2:**
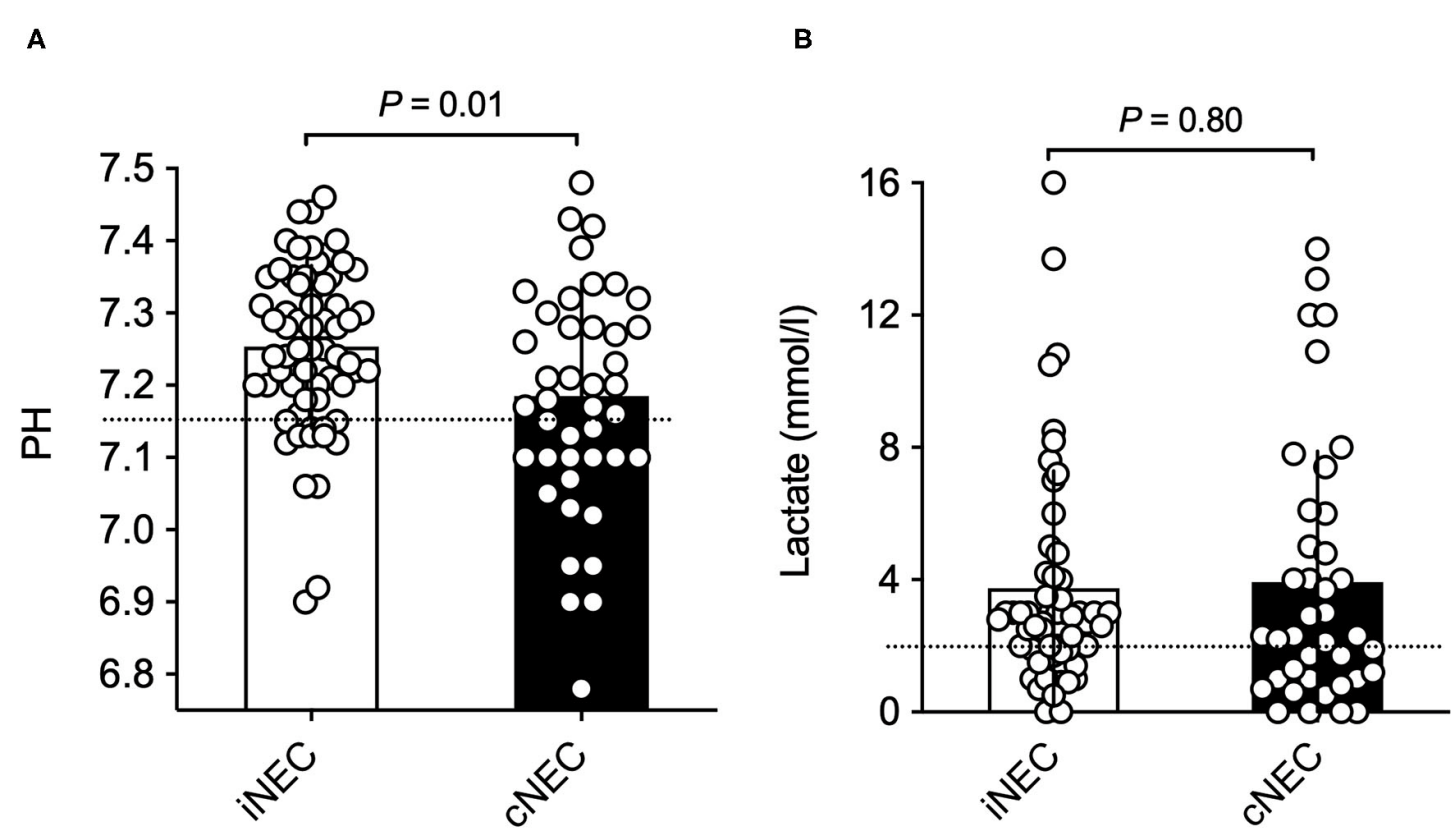
**(A)** Significantly increased leucocyte concentrations can be found in the cNEC group in comparison to the iNEC group prior to NEC surgery. **(B)** Neonates with cNEC demonstrate significantly increased neutrophil concentrations prior to confirmative NEC surgery in comparison to iNEC neonates.

**Figure 3:**
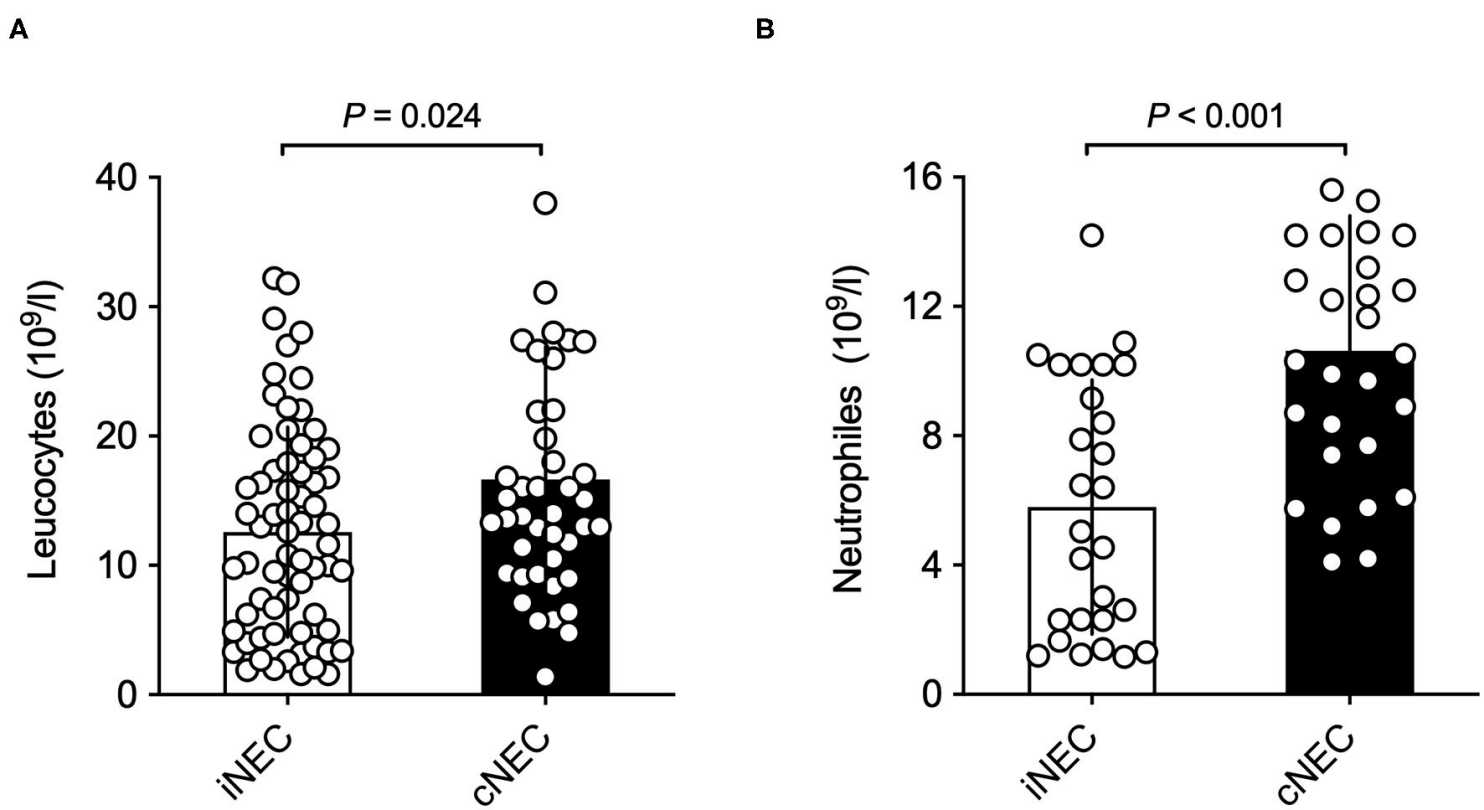
**(A)** Neonates in the cNEC group exhibit significantly decreased BGA pH values in comparison to patients in the iNEC group before NEC surgery has taken place. **(B)** No significant differences in BGA lactate levels prior to NEC surgery are found between NEC patients when stratified into cNEC and iNEC groups.

Significant differences were found with respect to laboratory values prior to surgically confirmed NEC diagnosis: Neonates within the cNEC groups demonstrated statistically significant increases in their leucocyte and neutrophil counts (Figure 2). The mean neutrophil concentration value for iNEC [5.79 (3.93)] was significantly lower than for cNEC [10.62 (4.18), *p* < 0.001, Figure 2B]. According to definition of Schmutz et al. 3.6% in the cNEC group but no patient with iNEC had neutrophilia (*p* = 0.52). Whereas, neutropenia was present in 38.5% in iNEC but never in cNEC patients (*p* < 0.001) (21). Blood-culture positive sepsis did not influence neutrophil levels [8.40 (5.11) vs. 8.22 (4.68), *p* = 0.92]. This finding is in accordance with the increase in leucocyte concentration observed within the cNEC group [16.64 (10.25)], which also is significantly increased when compared to leucocyte concentration values of the iNEC group [12.58 (5.11), *p* = 0.024, Figure 2A]. Lastly, with respect to laboratory parameters, both groups differed in their pH value prior to NEC surgery, as evaluated through BGA (Figure 3A). In fact, neonates assigned to the cNEC group on average showed significantly decreased pH levels [7.19 (0.16)] in comparison to the iNEC group [7.25 (0.11)] (*p* = 0.011), which, however, also experienced lower than average BGA pH levels (average 7.35–7.45). Upon further evaluation, BGA lactate levels between both groups did not differ significantly prior to NEC surgery and a sub-analysis revealed that only 55% of all included patients suffered from pathologically elevated lactate levels (>2), a potential marker of NEC severity and sepsis (lactic acidosis) as shown on Figure 3B. Moreover, in 15/67 (22.4%) of iNEC patients and in 7/43 (16.3%) cNEC positive blood cultures were found (blood-culture positive sepsis, *p* = 0.18).

Pneumatosis in US (cNEC 46% vs. iNEC 40%, *p* = 0.63) or Rx (cNEC 37% vs. iNEC 31%, *p* = 0.67) was present in less than half of the patients within 8 h before surgery that later revealed NEC. There were no significant differences between groups (Table 1). The other signs, like portal venous gas or free air were found even less frequently and there were no differences between cNEC and iNEC patients (Table 1). Ultimately, concerning all other characteristics or signs assessed in this study, no significant differences between both groups could be found (Table 1).

**Figure 4:**
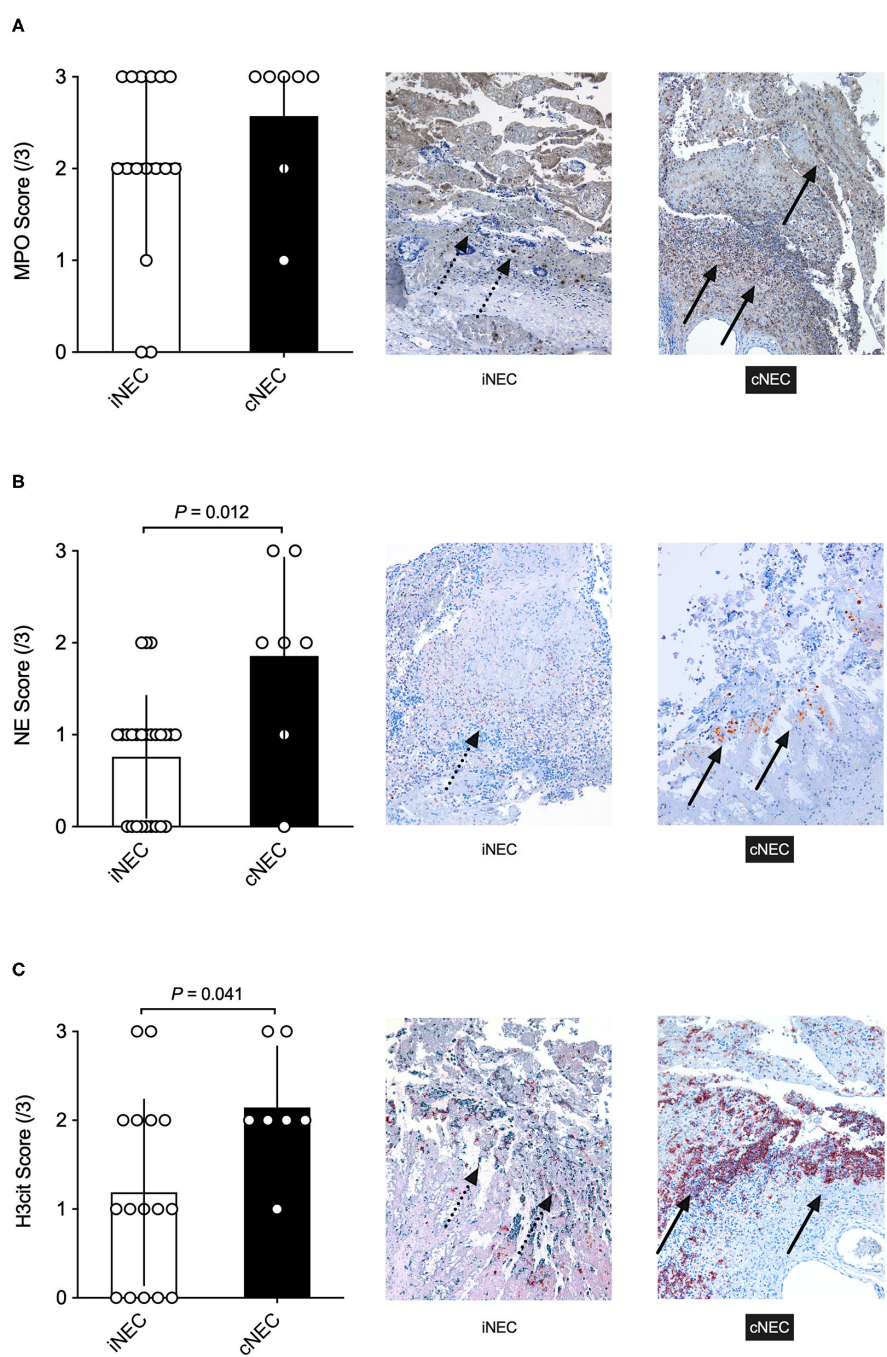
**(A)** No significant differences were found between the cNEC and iNEC groups with regards to MPO immunohistochemical staining and scoring of the affected intestine. **(B,C)** Neonates with cNEC demonstrate significantly increased NE and H3cit expression as analyzed through immunohistochemical staining of the NEC intestine in comparison to iNEC neonates. All images depicted are visualized using a magnification setting of 10x.

The study also conducted immunohistochemical staining of stored intestinal sample slides of NEC patients of the UKE and AKK during the time period assessed in this study, which were reanalyzed for MPO, NE, and H3cit (Figures 4A–C). As can be seen in Figure 4A, an increase, even though not significant, in staining and scoring for MPO slides within the cNEC group could be found in comparison to samples obtained from the iNEC group. Moreover, staining for NE showed a significant increase for neonates diagnosed with cNEC in comparison to neonates with iNEC (*p* = 0.012, Figure 4B), and neonates diagnosed with cNEC demonstrated significantly elevated H3cit levels in the intestinal tissue when compared to neonates in the iNEC group (*p* = 0.041, Figure 4C).

## Discussion

As previously described, NEC is an often-fatal disease affecting neonates and is associated with many severe short- and long-term consequences. As such, a thorough understanding of the pathomechanism of NEC to develop more effective diagnostic options, to prevent radical bowel resection surgery, is crucial (2). While this retrospective study sheds light on possible new diagnostic criteria for NEC, it also provides insights into the NEC pathomechanism and, with this, the option of novel treatment developments. The results suggest that NEC patient stratification into iNEC and cNEC, depending on cardiac comorbidities, might provide the ability to better diagnose NEC at an earlier stage and intervene sooner. Currently, NEC diagnosis relies heavily on the modified Bell Score, originally developed in 1978 to diagnose NEC in neonates, combining a myriad of (1) clinical symptoms, (2) laboratory parameters, and (3) radiographic criteria. Thus, this study aimed to assess most of the existing Bell Score criteria, while stratifying their prevalence based on the proposed cNEC and iNEC groups.

Upon stratification, the study revealed that neonates with cardiac comorbidities on average had higher neutrophil and, thus, leucocyte counts than iNEC patients directly prior to surgery. Bell et al. described neutropenia as a typical sign of NEC patients (22). In lieu of this, animal models have demonstrated that mice undergoing a NEC induction paradigm exhibit elevated neutrophil levels and their activity parameters, so that we hypothesize that neutrophils play a major role in NEC development (6). In line with this finding are the results from the immunohistochemical analysis of this study, which demonstrate that neutrophils are more prevalent in cNEC than iNEC intestinal samples, as measured by MPO, NE, and H3cit staining. Thus, a potential role of neutrophils in cNEC should be considered and markers of neutrophil activation and NETs formation should be evaluated as biomarkers of NEC in future studies.

One explanation for the differences in neutrophil and leucocyte concentrations among the two groups could be that iNEC's pathology is rather grounded in sepsis, while cNEC is based on ischemia reperfusion (I/R) injuries, which in turn is associated with classical neutrophilia and NETs formations (23–25). As drastically higher concentrations of neutrophils were found in cNEC compared to iNEC patients, it is certainly plausible that I/R injury due to cardiac comorbidities occurred, resulting in extreme neutrophil activation followed by a hyperinflammation reaction and NEC. Thus, the increase in neutrophil concentration in cNEC patients in our study might be explained by their role in mediating I/R injury. Based on the propositions that (1) infants with a cNEC experience a greater risk of intestinal I/R injury development and (2) I/R injuries play a significant role in NEC pathogenesis, then cNEC cases should exhibit a greater activity of the main mediators of I/R injury in blood, namely neutrophils (26). What is more, most cNEC patients in our study were diagnosed with a PDA, which as described earlier, is treated primarily with indomethacin at our clinic. On the other hand, various animal models have shown that indomethacin induces I/R injury of the gut, resulting in inflammation, thus further underlining our hypothesis of two different entities of NEC (27). Neutropenia is frequently observed in a proportion of preterm infants with neonatal sepsis and/or NEC (16.5–60%) and may result from insufficient numbers of circulating neutrophils combined with an immature neutrophil structure and function (28). Moreover, the innate immune system of prematurely born infants has been shown to be underdeveloped in comparison to term-born infants (29). Thus, given that the current study revealed that cNEC patients on average were older than neonates of the iNEC group at the time of NEC diagnosis, one might conclude, that younger NEC patients might be more prone of developing iNEC vs. cNEC due to the immaturity of the immune system and decreased neutrophil levels in response to combating infections. However, it appears as though both iNEC and cNEC involve neutrophils and especially NETs by inducing thrombosis, cytotoxicity and inflammation (20, 30).

Despite relatively unspecific clinical signs and symptoms for NEC—following have been identified: (1) feeding intolerance, mainly identified by bilious gastric retention; (2) abdominal distension; and (3) hematochezia. Neonates diagnosed with NEC at the AKK and UKE clinics during the study period frequently presented with abdominal distension followed by feeding intolerance, while all other signs were not indicative of NEC and extremely rare (31). Thus, many of the traditional textbook clinical signs of NEC appear to be unreliable markers for diagnostics of NEC development, which has also been reported in previous studies (22, 32). Moreover, cNEC and iNEC seem to share many signs and may not differentiated clinically. The age of the presentation (yet not corrected age), however, could assist in diagnosis, as cNEC patients present with NEC significantly later as shown by the current and previous studies (33, 34).

Another aspect of the modified Bell Scoring system for NEC diagnosis are imaging studies. As such, the main focus lies on abdominal XRs and US, in order to diagnose ileus, pneumatosis intestinalis, and pneumoperitoneum (22). However, despite particular radiographic presentations being indicative for surgery, imaging findings are often non-specific (35). These concerns are supported by our study, which demonstrates that no imaging investigation for NEC was able to accurately predict a future NEC diagnosis. Specifically, our study suggests that pneumatosis of the intestinal wall or portal venous gas are relatively rare in patients that were later confirmed to suffer from NEC. Additionally, no differences between the cNEC und iNEC group were found in regard to imaging studies and NEC development.

Finally, the last pillar in NEC diagnosis and to determine whether surgery is necessary, is the assessment of laboratory parameters. Again, the Bell criteria suggest that NEC development is a possibility when the patient of interest presents with either or all of the following: (1) metabolic (and/or respiratory) acidosis, (2) signs of disseminated intravascular coagulation (DIC), as indicated by thrombocytopenia, and/or (3) neutropenia (36). In addition, numerous other laboratory parameters aimed at diagnosing NEC during early stages have been assessed in recent years. Thus, our study has also focused on these laboratory parameters prior to NEC diagnosis in surgery.

With respect to acidosis, our study demonstrated a decrease in pH in both iNEC and cNEC patients, with the decrease being significantly lower in the cNEC group when compared to iNEC patients. However, lactate levels were only slightly increased in comparison to reference values and did not show any differences between both groups. More specifically, only 55% of all patients with confirmed NEC suffered from slightly elevated lactate levels. This result was particularly surprising as one would expect higher lactate levels in patients with inadequate (intestinal) microcirculation; i.e., the cNEC patients. However, due to the study design, many children that were considered cNEC patients had undergone PDA closure resulting in a possibly improved microcirculation within the eight-hour timeframe that was followed by surgery revealing NEC. That said, it appears that pH levels and not lactate should be used as a potential indicator for NEC, even though pH as such is highly unspecific. As most infants diagnosed with NEC receive ventilatory support one can assume that their pH is monitored continuously. Therefore, significant sudden decreases in pH should be an incentive for clinicians to further assess for NEC signs, especially if the neonate is receiving standardized ventilatory support.

Another parameter indicative of NEC according to Bell is the thrombocyte concentration as an indicator for DIC. In our retrospective analysis no significant differences between iNEC and cNEC patients were found and thrombocyte levels were not decreased in comparison to reference values of the normative patient population (37). One explanation of the normative platelet count in our study might be that the majority of neonates included in this study were Bell stage IIa/b and did not progress to NEC stage III or higher due to early surgical intervention, as thrombocytopenia has been shown to be associated with advanced NEC stages and adverse outcomes of treatment (38). Therefore, the use of thrombocyte levels as an early indicator for DIC in NEC might not be a reliable diagnostic criterion and should rather be used as a prognostic marker for NEC survival.

Prior studies have also analyzed the use of inflammatory markers, such as CRP, interleukins, and cytokine levels to diagnose NEC. While various cytokines such as IL-2, IL-4, IL-5, IL-6, IL-8, IL-10, INF-y have been associated with NEC (39, 40), the current study only assessed CRP and IL-6, as the other parameters are not routine diagnostics in our clinics. After analysis, our results showed that both NEC groups exhibited a rise in CRP and IL-6, yet no significant differences between both NEC groups existed. Regardless of the increase exhibited however, both IL-6 and CRP are very unspecific markers and can be elevated in a variety of different inflammatory diseases (41, 42). However, even though both parameters are non-specific for predicting NEC, the interplay of increased CRP, lactate, cytokines, and interleukins in combination with clinical signs and symptoms might be helpful in diagnosis and assessment of severity or outcome of NEC.

Even though the results of the study are promising, numerous limitations exist and need to be addressed. Firstly, the main limitation of the study is the retrospective design of the study, which makes controlling for possible confounders difficult, as well as establishing a true cause-and-effect relationship. However, the data analyzed in the study was extensive and documented electronically for all cases, making the temporal relationship between NEC diagnostic parameters and NEC diagnosis clear. Another potential limitation associated with the retrospective design of the study is the sample size. However, given the long time period of 10 years for data collection, a respectable sample size was obtained allowing for good reliability and decreased variability of results. Keeping this in mind, a larger and, preferably, multicenter study would allow for even more reliable and conclusive findings. Secondly, normal blood reference values for prematurely born children are rare due to the fact that most premature children exhibit a vast majority of pathologies, resulting in abnormal blood values. However, rough guidelines on blood parameters within the first weeks of life do exist, and were employed in this study (43). Thirdly, differing levels of neutrophils might be due to blood collection time coinciding with neutrophil transmigration from blood into the intestine (44). Thus, additional characterization of intestinal neutrophils in neonates with NEC would be of prime interest. Fourthly, as described above (1) neutrophil levels among neonates diagnosed with iNEC were significantly lower than those of patients with cNEC, and (2) patients with iNEC were significantly younger than patients stratified into the cNEC group. Thus, while we hypothesize that young NEC patients might be more prone for development of iNEC and not cNEC, one cannot assume a cause-and-effect relationship. The possibility that younger neonates cannot produce and/or activate neutrophils the same way as term-born neonates can and that a direct connection to the type of NEC development might not exist must be considered in this study. Therefore, the here observed younger patient age in the iNEC group might just be purely coincidental and based on the physiology of the developing innate immune system. Lastly, the current study did not assess ventilator settings, which were not documented for all patients. Different ventilation methods and settings could however affect neutrophil expression systemically and in the surrounding tissue. Hence, patients should be matched accordingly in future studies.

Overall, the study analyzed many different factors from clinical presentation and diagnostic imaging to laboratory parameters and confirms that early NEC identification is still challenging, as current laboratory and radiology tests lack sufficient discriminative power (45). Thus, there is a great need for more intrinsic research to find fast, cost-effective, and reliable markers of early stage NEC development. As, the results of this study, as well as previous animal model NEC research, suggest neutrophils' active role in NEC development, the use of NETs as a biomarker for cNEC development in order to avert fulminant disease progression should be assessed in future prospective studies.

## Data Availability Statement

The raw data supporting the conclusions of this article will be made available by the authors, without undue reservation.

## Ethics Statement

Ethical review and approval was not required for the study on human participants in accordance with the local legislation and institutional requirements. Written informed consent from the participants' legal guardian/next of kin was not required to participate in this study in accordance with the national legislation and the institutional requirements.

## Author Contributions

MK, HW, and DV designed the study, collected data, analyzed the data, and drafted and revised the paper. BB designed the study, collected data, and drafted and revised the paper. H-JS, LP, and KR collected data, and drafted and revised the paper. MB designed the study, collected data, analyzed the data, performed statistics and drafted and revised the paper. All authors contributed to the article and approved the submitted version.

## Conflict of Interest

The authors declare that the research was conducted in the absence of any commercial or financial relationships that could be construed as a potential conflict of interest.

## Abbreviations

NEC: necrotizing enterocolitis
NETs: neutrophil extracellular traps.
